# Co-morbidities to Vestibular Impairments—Some Concomitant Disorders in Young and Older Adults

**DOI:** 10.3389/fneur.2020.609928

**Published:** 2021-01-27

**Authors:** Eva-Maj Malmström, Eva Ekvall Hansson, Anna Hafström, Måns Magnusson, Per-Anders Fransson

**Affiliations:** ^1^Department of Clinical Sciences, Section of Otorhinolaryngology, Lund University, Lund, Sweden; ^2^Department of Neurosurgery and Pain Rehabilitation, Skåne University Hospital, Lund, Sweden; ^3^Department of Health Sciences, Lund University, Lund, Sweden

**Keywords:** dizziness, musculoskeletal pain, postural control, emotional strain, age

## Abstract

**Background:** Dizziness and pain are common complaints that often appear concomitantly, with or without a causal relationship. However, these symptoms might maintain and exacerbate each other and other co-morbidities. Therefore, adequate rehabilitation may have to include an expanded focus on other deficits and preconditions, especially in older adults and in patients.

**Objective:** To understand how frequently vestibular dysfunction coincided with medical conditions and aging, we studied two categories: Study 1: patients referred to a vestibular unit and Study 2: senior members in a fitness association.

**Method:** Study 1: 49 patients [34 females/15 males; mean age 52 years (SEM 2.0)] seeking health care for balance disorders and vestibular deficits were asked in questionnaires about their perception of dizziness and pain, and emotional and functional strains. Study 2: 101 senior members in a fitness association [91 females/10 males; mean age 75 years (SEM 0.6)], were assessed for vestibular and balance deficits and for any co-morbidities. The participants were monitored for falls for 12 months after the initial assessments.

**Result:** Study 1: Co-morbidity often existed between dizziness and pain (65%). The patients reported high emotional and functional strain related to their dizziness and pain. Patients older than 60 years reported longer durations of pain (*p* ≤ 0.028) but less emotional strain (*p* = 0.036), compared to younger patients. Study 2: 84% of the participants had a vestibular impairment, often without noticing any symptoms. Furthermore, 40% reported cardiovascular illnesses, 12% musculoskeletal disorders, and 63% reported other medical conditions. Forty-two percent experienced falls within 1 year after the initial assessments (thereof 42% in the group with vestibular deficits and 38% in the group without vestibular deficits).

**Conclusion:** To enhance and preserve postural control, both in patients with vestibular deficits and in older adults, we suggest an expanded clinical perspective. Hence, we recommend detailed examinations of the vestibular system but simultaneously probing for possible co-morbidities. Since aging often entails deterioration of multimodal processes related to maintained mobility and postural stability, our results add focus on the importance of addressing balance disorders together with additional medical conditions.

## Introduction

Remaining in equilibrium with adequate adjustments during locomotion and movement requires a constantly ongoing feedforward and feedback postural control system (1). This system uses a redundancy of sensory information from the vestibular organs, from proprioceptors, mechanoreceptors, and vision. These sensory inputs induce executive and adjusting motor responses throughout the entire body, based on the ability to adjust the individual segmental parts and the total body, and thus, maintain stability. The adjustments depend on both the biomechanical prerequisites as well as on well-functioning sensorimotor systems that produce controlled voluntary and automated movements through the body. Joint mobility, muscle strength and postural alignment together with cognitive perception and central processing merge into a function that needs to be able to address predictable and unpredictable requirements for maintaining balance and well-controlled movement (2–4).

The peripheral vestibular sensors can be considered as part of a multifaceted sensorimotor control system with several interacting and interfering entities. However, the normal aging processes cause over time increasingly larger multisensory deficits and multimodal deterioration within the postural control system, which impair the ability to produce accurate postural adjustments (5, 6). Some deficits can be compensated for, while others may remain to exacerbate and enhance dizziness or reduce physical function, and thereby cause loss of balance and in the end, falls (7–13). Moreover, recent reports has highlighted that pain may often be a co-morbidity to vestibular dysfunctions and in patients experiencing dizziness (14, 15).

A fall is an obvious and severe outcome of dizziness or loss of balance (16). The close dependence between musculoskeletal abilities and balance functions are essential in order to allow an active lifestyle with a preserved quality of life (16–18). As a consequence, balance deficits, dizziness, and pain often lead to a decrease in activities of daily living. Reduced activity relate to a frailty syndrome, which might lead to further loss of strength, mobility and physical agility (19). The co-existence of two or more chronic diseases has become more common with increasing age of the population, and hence, the need to further explore co-morbidities to design interventions (20).

These studies aimed to identify occurrences of some co-morbidities related to vestibular impairments and dizziness. More specifically, we wanted to investigate the co-existence of dizziness with other commonly occurring ailments, and especially with musculoskeletal pain. We also wanted to analyze how common vestibular dysfunction and impaired postural control (i.e., fall incidence) is in a population of still physically active older people.

## Materials and Methods

### Ethics Statement

The study conforms to the standards set by Declaration of Helsinki, 2004 and was approved by Regional Ethics Review Board (Study 1: Dnr LU178-07; Study 2: Dnr LU-2016/585), Lund University, Lund, Sweden. All subjects participated voluntary and provided written informed consent before taking part in the study. The data collection used in study 1 is an extraction from an already published article from our research group, the extracted data here categorized and analyzed to determine co-morbidities (15).

### Study 1: Co-morbidities in Patients Referred to a Vestibular Unit

#### Subjects

The study included 49 patients [34 females/15 males; mean age 52.0 years (Standard Error of Mean (SEM)) 2.0], consecutively referred to a tertiary referral center for examination of vestibular deficits and balance dysfunction. To be included the patients had to be adult, conceding to be a part of the study, being able to understand and communicate, and fill out the required questionnaires. The patients thus had a variety of diagnosis with symptoms of dizziness or perceived balance impairment. Study questionnaires were sent to the patients 2 weeks before the visit and collected at the visit. Patients that subsequently failed to return the questionnaire were excluded. The population was divided into three subgroups for comparisons; (1) subjects ≥60 years of age (*n* = 15); (2) subjects <60 years of age (*n* = 34), and (3) the youngest 15 subjects in the population (*n* = 15). Results from the total study population has been partly reported, with allowance of extraction for the purpose of the present paper (15).

#### Test Procedure

The patients answered two questionnaires, the Dizziness Handicap Inventory (DHI) questionnaire and a custom-made questionnaire detailing the properties of pain, dizziness, and occurrence of trauma/accident, considered by the patient to be related to their symptoms.

##### The Dizziness Handicap Inventory Questionnaire

This questionnaire included 25 questions related to the dizzy symptoms, assessing perceived handicap and impact on quality of life (21–25). In the study, a validated version of the DHI questionnaire in Swedish was used (22). The DHI results can both be presented as “Total” (the sum of all question scores), with a maximum scored interference at 100 (questions answered with a “Yes” —scored 4, “Sometimes” —scored 2, and “No” —scored 0). The DHI questionnaire can also be divided into three symptom domains (“Physical,” “Emotional,” and “Functional”) (21).

##### The Pain and Dizziness Questionnaire

This questionnaire comprised eight questions, that queried intensity and duration factors related to dizziness and if present, of a pain (Table 1) (15). For symptoms, the patients reported intensity and severity using an 11 point Numeric Rating Scale (NRS) (26, 27) (0 = no symptoms, 10 = most severe symptoms imaginable). If the patients reported pain, questions were also asked about interference on activities (“severity”) and locations for their pain (head, including headache; neck/shoulders; back; arms; upper torso; lower torso; legs and feet). If the patients reported pain, they were asked for any potential history of accident/trauma they considered to be related with the experienced pain.

**Table 1:** Pain and dizziness and accident questionnaire.

**Question**	
1^a^	How long have you experienced dizziness (months)?
2^b^	Dizziness intensity – How severe is the dizziness on average?
3^c^	Do you feel pain or tension in your body?
4	How long have you experienced pain (months)?
5^d^	Accident event – Have you been involved in any accident/trauma you consider caused the pain?
6^b^	Pain intensity – How severe is the pain on average?
7^b^	Pain severity – How severely has the pain affected your activities the last 24 h?
8	Pain distribution – Mark the locations where you experience pain Head □, Neck/Shoulder □, Back □, Arms □, Upper torso □, Lower torso □, Legs □, Feet □

a * Reporting Dizziness = Answering with a value >0 on question 1 or question 2*.

b *Graded by Numeric Rating Scale [0 … 10]*.

c *Reporting Pain = Answering Yes on question 3*.

d *Reporting Accident event = Answering Yes on question 5*.

### Study 2: Co-morbidities in Physically Active Older People With or Without Vestibular Deficits

#### Subjects

The participants were senior members of a Swedish fitness association who were invited to a balance workshop. The workshop was repeated at three different times to allow all volunteering subjects to attend. The inclusion criterion was that the subject should be an active participant in a fitness association, and by that indirectly, having an independent and physically active lifestyle. Subjects that failed to return the fall incident dairies during the 12-months observation period were excluded. In total, 150 persons attended the workshops. Out of these, 113 accepted to participate in the study. The 101 participants [91 females/10 males; mean age 75.0 years (SEM 0.6)] who performed all testes and returned at least one fall diary were included in this study. After performing a series of vestibular assessments the population was divided into two categories, subjects with vestibular deficits (*n* = 85) and subjects without vestibular deficits (*n* = 16). The subjects in the two categories had similar mean age (75.1 vs. 74.8 years) and both categories were dominated by females (**Table 5**).

#### Procedure

Baseline information about age, gender, illnesses, and medication was self-reported. Vestibular function was assessed by a specialist in neuro-otology (author MM) with the headshake test, Dix-Hallpike test (28), and head impulse test (29, 30). The Timed up and Go test (TUG), Timed up and Go test with a cognitive task (TUGcog), and Timed Up and Go test with a manual task (TUGman) (31) were used for measuring balance. The performance of the tasks was assessed by a registered physiotherapist recording the time (author EEH).

##### Vestibular Tests

Three screening tests of the vestibular function were performed (32). The eye movements were recorded with a Video Nystagmoscope from Synapsis™ during all vestibular tests. The responses were considered pathologic if nystagmus beats occurred (headshake, Dix-Hallpike tests) or a saccade occurred (head impulse test) (33).

Vestibular asymmetry was assessed by the headshake test, where the tested person was lying supine and the examiner shook their head from side to side for 15 s at ~2 Hz.

Benign paroxysmal positional vertigo for the posterior and anterior canals were assessed by the Dix-Hallpike test. The tested person sat on an examination table, with their head turned about 45° to the left or right. The examiner thereafter moved the patient down into a supine position, with the patient's supported head leaning back about 30 degrees. The test was considered positive if nystagmus and vertigo occurred (28).

For assessing hypofunction in the angular vestibulo-ocular reflex, the head impulse test was used. The tested person was asked to focus the gaze on the examiner's nose, and the examiner rotated the patient's head slowly from side to side. The examiner then turned the head rapidly about 20° to one side. The test was repeated randomly to each side. The test was considered pathologic if the tested person was not able to keep their eyes focused on the target, but responded with a corrective or compensatory saccade (29, 30).

##### Balance Measures

Balance was assessed by the TUG, TUGcog, and TUGman. In TUG, the tested person was instructed to stand up from a chair, walk in a fast but safely pace three meters and cross a line marked on the floor, turn around, walk back to the chair, and sit down (31). In TUGman, the tested person was instructed to grab a glass filled with water when standing up, walk the three meters as in TUG and put the glass back before sitting down. In TUGcog, the tested person was instructed to count aloud numbers down from 100 with 3-number intervals during the tests (34). The performance of all TUG tasks were rated by the time required to complete the task sequences.

##### Falls

All participants were given a falls diary booklet at the workshop, in which the participant noted falls during a 12-months period. The participants were instructed to detail in the diary the number of times they had fallen and describe the situational characteristics of the incidents associated with the falls. Every third month during the 12-months follow-up, a new diary was sent home by ordinary mail, together with a pre-paid envelope, which the participant used for returning the filled-in diary. The falls was categorized according to a classification system from St. Louis, Older Adult Service and Information System (OASIS) (35).

### Statistical Analysis

#### Study 1

Our initial analysis revealed that patients referred to a vestibular unit often displayed symptoms that seemed related to their age above and below 60 years of age. Therefore, to reveal better any age effects, data from the subjects ≥60 years were compared with all subjects below 60 years of age, and a subgroup of the 15 youngest subjects in the population. Moreover, pain in various parts of the body was also very common and the patients reported that their symptoms might be associated with an accident they had experienced (Table 1). The roles of these three potential co-morbidities factors were evaluated individually with either Mann-Whitney *U* (two-tailed) analysis or the Pearson's Chi-square test when appropriate (36). In the analyses, p-values <0.05 were considered statistically significant (36). P-values to the level of trends (*p* < 0.1) are presented in the figures and marked with bold text in the tables.

Using the statistical package GPower 3.1™, sample size analyses of parameter categories, such as DHI revealed an effect size of 1.1, which shows that with the *p*-value set to 0.05 (two-tailed), our study would require *n* = 14 subjects to reach a power value of 0.8 for this parameter category (36). The sample size analysis of the pain distribution parameters revealed an effect size of 1.4, which shows that with the *p*-value set to 0.05 (two-tailed), our study would require *n* = 10 subjects to reach a power value of 0.8 for this parameter.

#### Study 2

The between-groups comparisons were made with either Mann-Whitney *U* (two-tailed) analysis or the Pearson's Chi-square test when appropriate (36).

Non-parametric statistical methods were used since distribution tests with Kolmogorov-Smirnov and Shapiro-Wilk methods revealed that some of the datasets did not have a normal distribution profile and that normal distribution could not be obtained by logarithmic transformation. In the analyses, *p*-values <0.05 were considered statistically significant (36). Bonferroni correction was applied but had no practical effect as all datasets in the statistical between-groups evaluations were included only once in a comparison. P-values to the level of trends (*p* < 0.1) are marked with bold text in the table.

In study 2, measures of step time was also performed, using a wearable device. Step time was used for calculation of power. Expecting 50% more falls among persons with a variance in step time of ≥2.0 ms, and the level of significance set at 0.05, a total of 60 participants would be necessary to reach a power of 80%.

The statistical analyses were performed with SPSS 26.0 software (SPSS Inc., Chicago, IL, USA) and the power analysis was performed with GPower 3.1™.

## Results

### Study 1: Co-morbidities in Patients Referred to a Vestibular Unit

The patients with balance disorders showed signs of physical, emotional and functional strain, irrespectively of age. In the total DHI score, 19 of 49 subjects (39%) reported severe handicap; 9 of 49 subjects (18%) reported moderate handicap, whereas 14 of 49 subjects (29%) reported mild handicap. A large proportion of the patients, 65.3% (SEM 6.9), presented co-morbidity with pain to the levels that it influenced daily life. Another co-morbidity factor was that 24.5% (SEM 6.2) of the subjects reported that they believed some of the symptoms they experienced were related to a history of an accident.

#### Evaluation of Differences Between Age Populations

Females were overrepresented in all age categories, but the gender distribution was not significantly different between any of the categories (Table 2). The older population tended to score lower in the emotional subscale in the DHI questionnaire than the <60 years category (*p* = 0.054), and significantly lower than the youngest age category (*p* = 0.036), suggesting less emotional strain. Pain symptoms were commonly reported in all populations and this did not differ significantly between the older and younger populations. However, the older population reported significantly longer pain duration than the <60 years category (*p* = 0.023), and the youngest age category (*p* = 0.028). Finally, the dizziness duration tended to be longer in the older population than in the youngest population (*p* = 0.063).

**Table 2:** Characteristics and questionnaire results for the patients, divided by age.

**Material** ** characteristics**		**Population^a^**			**Statistics^b^**	
		**Aged ≥0 years**	**Aged <60 years**	**15 youngest subjects**	**Aged ≥60 years vs. Aged <60 years**	**Aged ≥60 years vs. 15 youngest subjects**
Gender		11f/4m	23f/11m	9f/6m	0.691	0.439
Age (years)		68.5 (1.7)	44.8 (1.5)	36.5 (1.3)	–	–
Dizziness duration (months)		110.1 (31)	52.6 (12.6)	35.4 (9.8)	0.121	**0.063**
Dizziness intensity^c^		5.5 (0.4)	6.3 (0.5)	6.2 (0.7)	0.276	0.315
DHI^d^	Total	37.5 (6.4)	43.9 (3.9)	43.3 (4.4)	0.356	0.383
	Physical	12.8 (1.8)	11.6 (1.3)	10.7 (1.5)	0.609	0.504
	Emotional	11.7 (2.5)	16.9 (1.5)	18.1 (2.1)	**0.054**	**0.036**
	Functional	13.0 (2.6)	15.5 (1.6)	14.5 (2.0)	0.339	0.506
Reporting pain (%)^e^		60.0 (13.1)	67.6 (8.1)	73.3 (11.8)	0.604	0.439
Pain duration (months)		175.2 (21)	68.7 (13)	41.6 (13.5)	**0.023**	**0.028**
Pain intensity^c^^,^^e^		5.8 (0.5)	5.3 (0.4)	4.1 (0.6)	0.604	0.114
Pain severity^c^^,^^e^		5.3 (0.7)	6.2 (0.5)	5.4 (0.8)	0.631	0.795
Reporting accident (%)		20.0 (10.7)	26.5 (7.7)	26.7 (11.8)	0.627	0.666

a *The values are presented as mean and SEM-values, the latter presented within the parenthesis*.

b *The notation “ <0.001” means that the p-value is smaller than 0.001*.

c *Scaled as 0 = no symptoms and 10 = most severe symptoms imaginable*.

d *DHI represent the values obtained from the Dizziness Handicap Inventory questionnaire*.

e *Pain represent whether the patients reported pain anywhere at the eight locations detailed*.

##### Pain Distribution When Sorted in Different Factor Categories

**Figure 1:**
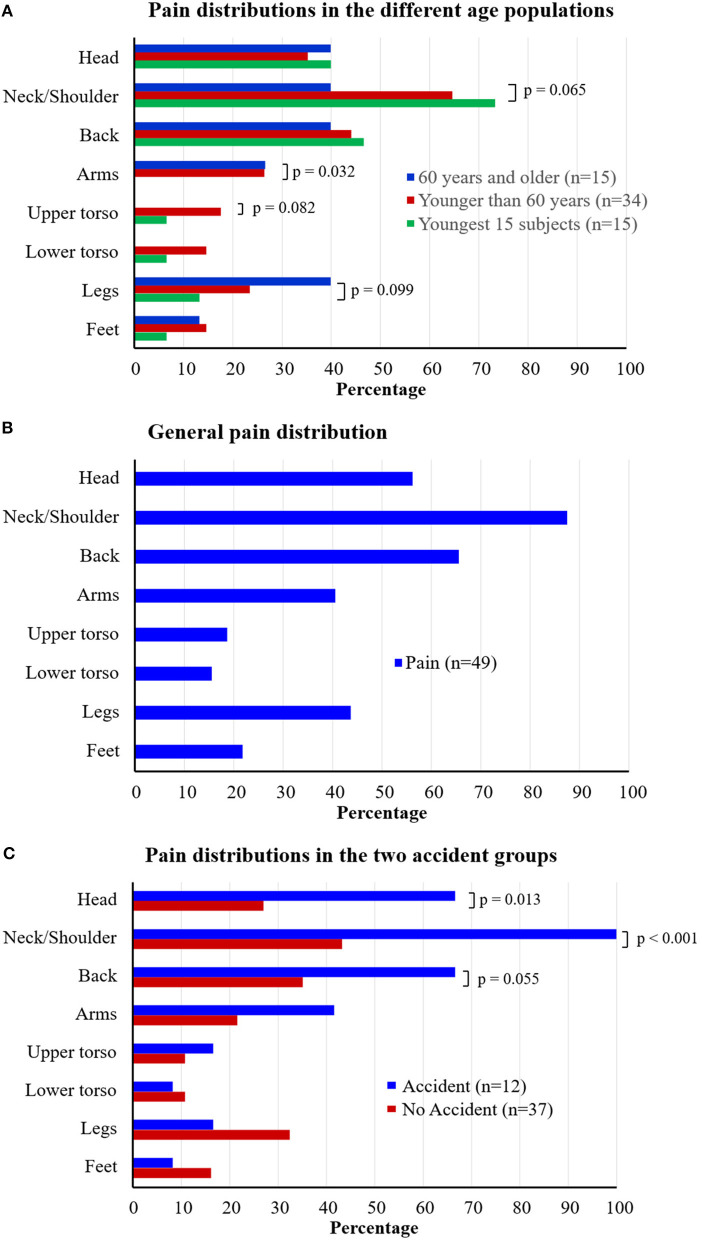
Pain distribution patterns for: **(A)** each age population; **(B)** among all patients reporting pain; **(C)** the patients reporting a history of an accident and not having a history of an accident. The values on the x-axis denotes the percentage of the patients reporting pain with the pain localization on the y-axis. Note that a patient can report pain in more than one location. *P*-values to the level of trend <0.1 are detailed.

The population <60 years tended to report pain more often in the upper torso region compared with the older population (Figure 1A), though not reaching significant levels (*p* = 0.082). Moreover, the youngest population tended to report more pain in the neck/shoulder region (*p* = 0.065), but significantly less pain in the arms (*p* = 0.032) and also a tendency of less pain in the legs (*p* = 0.099) than the older population.

#### Evaluation of Differences Between Patients With and Without Pain

The females suffered more often from pain than males (*p* = 0.013) (Table 3). The patients with reported pain scored significantly higher in the DHI questionnaire, both in Total (*p* = 0.004) and in the DHI sub-scales DHI_Emotional (*p* < 0.001) and DHI_Functional (*p* = 0.011). A significant number of the patients that reported pain had a history of an accident (*p* = 0.004).

**Table 3:** Characteristics and questionnaire results for the patients, divided by pain symptoms.

**Material** ** characteristics**		**Symptoms^a^**		**Statistics**
		**Pain^b^**	**No pain^b^**	***p*-Values^c^**
Gender		26f/6m	8f/9m	**0.013**
Age (years)		50.7 (2.5)	54.5 (3.2)	0.488
Dizziness duration (months)		86.3 (19)	34.3 (9)	0.146
Dizziness intensity^d^		6.3 (0.4)	5.5 (0.7)	0.280
DHI^e^	Total	48.7 (3.9)	29.3 (5.0)	**0.004**
	Physical	12.9 (1.3)	10.1 (1.5)	0.192
	Emotional	18.5 (1.5)	9.2 (1.9)	** <0.001**
	Functional	17.2 (1.6)	10.0 (2.2)	**0.011**
Pain duration (months)		95.4 (16)		
Pain intensity^b^^,^^d^		5.4 (0.4)	–	–
Pain severity^b^^,^^d^		5.9 (0.5)	–	–
Reporting accident (%)		37.5 (8.7)	0.0 (0.0)	**0.004**

a * The values are presented as mean and SEM-values, the latter presented within the parenthesis*.

b *Pain represent whether the patients reported pain anywhere at the eight locations detailed*.

c *The notation “ <0.001” means that the p-value is smaller than 0.001*.

d *Scaled as 0 = no symptoms and 10 = most severe symptoms imaginable*.

e *DHI represent the values obtained from the Dizziness Handicap Inventory questionnaire*.

##### Pain Distribution When Sorted in Different Factor Categories

In the patients suffering from any kind of pain, the most common location for pain were the head (56.3%), the neck/shoulder (87.5%), the back (65.6%), and the legs (43.8%) (Figure 1B).

#### Evaluation of Differences Between Patients With and Without a History of an Accident

Females tended to more often have experienced an accident than males (*p* = 0.054) (Table 4). The patients who reported a history of an accident scored not significantly different than the non-accident patients in the DHI questionnaire. However, the patients in the accident group suffered significantly more often from pain than the patients in the no accident group (*p* = 0.004).

**Table 4:** Characteristics and questionnaire results for the two accident groups.

**Material** ** characteristics**		**Symptoms^a^**		**Statistics**
		**Accident**	**No accident**	***p*-Values^b^**
Gender		11f/1m	23f/14m	**0.054**
Age (years)		50.1 (3.6)	52.6 (2.4)	0.658
Dizziness duration (months)		83.3 (23)	65.1 (16)	0.179
Dizziness intensity^c^		5.8 (0.8)	6.1 (0.4)	0.736
DHI^d^	Total	48.8 (6.6)	39.7 (3.8)	0.285
	Physical	12.2 (2.4)	11.9 (1.1)	0.916
	Emotional	18.3 (2.5)	14.3 (1.6)	0.217
	Functional	18.4 (2.6)	13.5 (1.6)	0.124
Reporting pain (%)^e^		100.0 (0.0)	54.1 (8.3)	**0.004**
Pain duration (months)		110.1 (27)	83.3 (14)	0.447
Pain intensity^c^^,^^e^		5.3 (0.6)	5.5 (0.4)	0.823
Pain severity^c^^,^^e^		6.0 (0.8)	5.9 (0.4)	0.951

a * The values are presented as mean and SEM-values, the latter presented within the parenthesis*.

b *The notation “ <0.001” means that the p-value is smaller than 0.001*.

c *Scaled as 0 = no symptoms and 10 = most severe symptoms imaginable*.

d *DHI represent the values obtained from the Dizziness Handicap Inventory questionnaire*.

e *Pain represent whether the patients reported pain anywhere at the eight locations detailed*.

##### Pain Distribution When Sorted in Different Factor Categories

The patients reporting that they had an accident in their history had significantly more often pain in the head (*p* = 0.013) and neck/shoulder (*p* < 0.001) and tended to have more often pain in the back (*p* = 0.055) compared with patients that had no history of an accident (Figure 1C).

### Study 2: Co-morbidities in Physically Active Older People With or Without Vestibular Deficits

#### Clinical Findings

After excluding participants with incomplete baseline measurements and participants who did not return a complete set of fall-diaries, a total number of 101 participants were included in the statistical analysis. In the population category that had vestibular deficits (*n* = 85), most of the subjects had pathological headshake nystagmus (90.6%) and pathological head impulse responses (46.4%) (Table 5). A smaller group also had pathological Dix-Hallpike responses (22.4%). Note that subjects can present pathological responses in several of the vestibular tests.

**Table 5:** Characteristics for the two vestibular groups.

**Material** ** characteristics**		**Symptoms^a^**		**Statistics**
		**Vestibular pathologies**	**No vestibular pathologies**	***p*-Values**
Gender		78f/7m	13f/3m	0.196
Age (years)		75.1 (0.6)	74.8 (1.6)	0.841
Medical conditions (%)		67.1 (5.1)	62.5 (12.5)	0.723
Cardiovascular illness (%)		38.8 (5.3)	43.8 (12.8)	0.712
Musculoskeletal disorder (%)		10.6 (3.4)	18.8 (10.l)	0.355
Other illnesses (%)		28.2 (4.9)	12.5 (8.5)	0.187
More than one illness (%)		14.1 (4.2)	18.8 (10.1)	0.687
Detection methods	Headshake^b^ (%)	90.6 (3.2)	–	–
	Dix-Hallpike^b^ (%)	22.4 (4.5)	–	–
	Head impulse^b^ (%)	46.4 (5.4)	–	–
Balance	TUG^c^ (s)	8.5 (0.2)	8.3 (0.3)	0.696
	TUGman^d^ (s)	9.4 (0.2)	9.2 (0.4)	0.900
	TUGcog^e^ (s)	10.2 (0.2)	10.2 (0.3)	0.820
	Difference TUGman-TUG (s)	1.0 (0.2)	0.9 (0.3)	0.659
	Difference TUGcog-TUG (s)	1.8 (0.1)	1.9 (0.3)	0.602
Reporting falls (%)		42.4 (5.4)	37.5 (12.5)	0.718
Number of falls classified as extrinsic/intrinsic		30/9	15/2	0.230

a * The values are presented as mean and SEM-values, the latter presented within the parenthesis*.

b *The subjects can present pathological responses simultaneously in several of the vestibular tests made*.

c *TUG, Timed up and Go test*.

d *TUGman, Timed up and Go test with a manual task*.

e *TUGcog, Timed up and Go test with a cognitive task*.

Even if many of participants reported that they had no illnesses, many of them used some kind of medication. Therefore, the categorization of illnesses was based on both reported illness and reported medication. In the total population, 40% reported cardiovascular illnesses, 12% musculoskeletal disorders, and 63% reported other types of illnesses that affected daily life. The frequencies of illnesses were very similar in both categories of vestibular deficits and without vestibular deficits. Cardiovascular illnesses were common in both categories, 38.8 vs. 43.8%. Severe musculoskeletal disorders and other illnesses were also common in both categories (10.6 vs. 18.8%) resp. (28.2 vs. 12.5%). Some subjects in both categories reported more than one illness (14.1 vs. 18.8%).

The mean values and standard error of mean for age, the TUG, TUGman, and TUGcog tests and the results for health status and vestibular tests are shown in Table 5. The age, gender, health status, and stability as reflected by results in the functional tests were not significantly different between subjects with vestibular deficits and subjects without deficits. During the 12-months follow up, 42 participants reported one or more falls, and a total number of 58 falls. The majority of falls was categorized as extrinsic falls (77%), caused by perturbed stance, perturbed gait, or external cause of loss of balance. Almost one fifth of the falls was categorized as intrinsic falls (19%), caused by vertigo, legs giving away, or loss of postural control. A few falls was not classifiable (4%). There was no difference in age, cardiovascular illnesses, musculoskeletal disorders, or vestibular status between fallers and non-fallers.

## Discussion

We found that patients with dizziness also commonly report musculoskeletal pain and that co-morbidities between dizziness and pain add psychological distress. In addition, older subjects, healthy enough to participate in physical activities, frequently had asymptomatic vestibular deficits. Almost half of these physically active older people experienced one or more fall incidence during the following year.

### Co-morbidity Between Dizziness and Pain

Although both dizziness and pain are common in a general population, the concurrent appearance of these two symptoms might suggest an interrelationship as well as an additional burden for the individual. The patients with pain demonstrated significantly higher levels of perceived handicap of their dizziness, and thus, that this had consequences on their perceived quality of life compared to patients that did not report pain. Older patients stated significantly longer pain duration and they reported more pain in their lower extremities compared to the younger population, who had more pain in the neck/shoulder and torso regions (Figure 1).

In patients that reported pain, 38% also reported a history of an accident the patient themselves thought was the origin of the pain. These patients had significantly more often issues with pain in the head and neck/shoulder regions and reported more often back pain (Figure 1C). Pain in these segments has been shown to affect the sensorimotor control (37). Moreover, especially after neck trauma, pain in these specific segments are known to be associated with dizziness (38). The intersegmental coordination between different parts of the body changes with age and disturbances of the cervical proprioceptive information especially seems to disturb and change postural strategies (6). This emphasizes the importance of reducing pain and disability from the different body regions in order to make intersegmental coordination as optimal as possible, also in dizzy patients.

The high frequency of reported pain conditions in lower extremities, the neck and the shoulder regions in this study group of dizzy patients, highlight the importance of asking patients about any pain conditions and history of trauma.

### Age-Related Multimodal Decline in Sensorimotor Systems and Comorbidities

Since aging often entails deterioration of multisensory and multimodal processes related to maintained mobility and postural stability, our results add focus on the importance of paying attention and taking direct actions when balance disorders occur together with pain and other medical conditions. Especially when dizziness occurs together with pain in the neck/shoulder regions and in the lower extremities, the symptoms might interact and maintain or even enhance each other. To some extent, the balance deficits may be compensated for, but the adjustment options might be fewer due to pain related impairments. Thus, the functional loss may become more prominent, with reduced physical function and thereby also a loss of postural stability (7–13).

Pain in the lower extremities is often related to reduced muscle strength, which *per se* is regarded as one important factor for preserved postural stability (7). In addition, mobility and proprioception that also might be affected by pain are of uttermost importance to retain postural stability during upright stance and locomotion (9, 33, 39). Here, the ankle mobility, the mechanoreceptors of the feet, and also the proprioceptors of the intertarsal muscles is of special interest, keeping us stable with adequate reactive response relative to the surface (9). Because proprioceptive information often is also decreased in older people without pain, a reduction of these sensory inputs might become even clearer in situations that demand postural stability (40). A rigid strategy caused by either pain or dizziness, or both simultaneously, might also affect intersegmental mobility and coordination. Here, older people already seem to have a different coordination strategy (6). Older people also seem to have a different postural strategy compared to younger, including less effective adaptive processes to address stability issues (10). Especially in the frontal plane, older people seem to adapt to postural disturbances in a less appropriate way than younger subjects (41), a factor that can be negatively additive to an already present pain condition in the lower extremities. With the clear importance of adequate proprioceptive input, muscular strength and coordination and good mobility, for appropriate postural responses and thereby maintained postural stability, postural control is considerably and indisputably multisensory, multifactorial and multimodal. Therefore, pain conditions and biomechanical prerequisites should be considered and examined in parallel with proper vestibular examinations when balance problems are being addressed and treated, irrespectively of age.

### Psychological Distress of Co-morbidity Between Dizziness and Pain

The patients reporting both dizziness and pain showed significantly more psychological distress, demonstrated by higher scoring values on questions associated with emotional status in the DHI questionnaire (24). While high psychological distress was reported for the patients with pain, compared to younger subjects the older subjects reported somewhat less impact on the emotional domain (Table 2). This is consistent with results showing a negative correlation for emotional distress with age suggesting possibilities for adaptation of daily activities and thereby an increased sense of control in older, retired people (42). Still, older subjects reported high scores in the DHI questionnaire, suggesting a considerable emotional load anyway and interference of daily activities. Hence, the emotional factor is a substantial handicap to consider also among older adults (23, 43, 44).

### Physically Active Older Adults With Vestibular Deficits and Fall Incidents

The majority (84%) of the older subjects active in a fitness association demonstrated vestibular asymmetry or other balance function disturbances. Most of them were not aware of such dysfunctions. That said, the study participants all had responded to an invitation to take part in a workshop about balance, which might suggest that at least some of them had a specific interest in such a topic. Thus, one may speculate that this interest might be instigated by them having experienced instability and balance disturbance. If that was the case, that would explain the proportionally very high amounts of vestibular asymmetry in the studied group compared to other populations (5, 8, 45, 46). On the other hand, the participants considered themselves healthy, were active in a fitness association, and performed well in the TUG tests. This speculative view stands in contrast to that almost half of the whole group had sustained falls at the 1-year follow-up. However, a large majority of these falls (77%) were by the participants regarded to be caused by extrinsic factors. Furthermore, the subjects with identified vestibular pathologies did not fall more often compared to those without such disorders.

More than half of the group reported impairment related to other medical conditions, which highlight the importance to evaluate the person's entire state of health regarding their ability to maintain postural control. Vestibular dysfunctions are common in the older population and together with physiological changes and multi-morbidity it implies a risk of hazardous events, such as falls (12). Kristinsdottir et al. have demonstrated that vestibular asymmetries could contribute to falls and thereby even fractures in older people (47). Our results demonstrate that falls among older subjects are so common that specific balance training programs could be suitable for all, especially since such programs have shown to affect vestibular asymmetry positively (46). In addition, more than half the group had other declared illnesses, such as diabetes and high blood pressure, which also may be suspected to affect balance control and requiring specific attention of the primary health care. Low blood pressure is also a common issue among older people, but none of the participants in our studies reported taking any medications that could be related to treatment of low blood pressure. Hence, addressing balance problems to older persons visiting primary health care and providing specific balance programs, including vestibular rehabilitation, might decrease the risk of falls and should be offered not only to persons with severe balance problems.

### Limitations

Both sub-studies were explorative, carried out independently and performed in two different contexts. However, they both addressed co-morbidities to vestibular impairments and dizziness.

Both study 1 and study 2 included subjective self-reported data. Thus, in study 1 questionnaire answers and reports of pain might be skewed by, e.g., subjective exaggeration of symptom severity. However, a recently published study with similar approach and scope as in study 1 largely confirm our findings (14). Study 2 included physically active subjects. Even if they suffered from conditions linked to aging or chronic diseases, most did not perceive themselves as being impaired. This may reflect a mental state of habituation and thus, there is a risk that co-morbidities were under-reported in study 2. Therefore, subjects were asked about medications. If medications suggested a chronic disease, such co-morbidity was considered present even if the participant did not report it initially.

In study 2, the commonly used and simple to perform TUG test was utilized to make a primary assessment of postural control, as some studies suggests that this method can determine fallers from non-fallers (48, 49). An optimal cut-off value for TUG in predicting future falls among community-dwelling older persons has been suggested to be 12.6 s (48). However, this value is much higher than we recorded in both participants with and without vestibular deficits in our study. Thus, TUG does not seem to be optimal for predicting falls among older persons who considered themselves as healthy and that regularly are physically active, in our case as part of a membership in a fitness association. This difficulty to predict falls with tests has lately been demonstrated by Bobowik et al. (49). A possible explanation to why the falls still were so common in the population category might be that the falls often (77%) were deemed caused by extrinsic factors like tripping and slipping, i.e., when the subject is exposed to external demands for which there are not sufficient resources. The aims and scope of study 2 was limited to determining if vestibular deficits and a limited set of various co-morbidities were common in a physically active cohort of older people subjects, and if so, if these factors had a substantial effect on their postural control. One reason for this approach was to determine if specific vestibular deficits were over-represented among fallers. This turned out not to be the case. Thus, falls can be caused by a number of factors, and it would be interesting if these factors could be detailed in future studies, for example, by using more advanced methods to assess postural control and by screening the study participants for more kinds of relevant medical conditions.

### Clinical Implications

The findings highlight some of the most important factors for our ability to be active and in good health at old age. A good body balance, good postural alignment, and preserved muscle strength are considered to be protective factors for maintain an active lifestyle in aging. These factors also coincide with good quality of life (17). Since most stability issues originating from vestibular and other sensorimotor deficits can be addressed, or at least ameliorated, it is critical that they are detected by the health care system (46, 50). The interventions to enhance and maintain postural control during aging should preferably be multimodal, able to detect and address co-morbidities, such as pain and mental strain, and performed in a structured but still adjustable, customized way (51). Both rehabilitation and training in general for older people have to meet the impairments that are common in dizzy patients and in older people in general.

In summary, based on the clinical implications of our results, together with previously acquired knowledge, we recommend health care practitioners to always ask for, and consider the co-morbidities that often coincide with dizziness. Pain should be considered a common co-morbidity, of importance to address both for optimal vestibular rehabilitation and for training postural control. Having a history of an accident/trauma may also be a contributing factor to dizziness, pain, and sensorimotor deficits. When performing rehabilitation, the exercises should promote a holistic approach of maintaining healthy muscular and biomechanical function in all intersegmental levels, as well as to facilitate optimum use of integrated sensory information from all sensory sources, i.e., vision, vestibular, mechanoreceptors, and proprioceptors. Older adults may be more prone to suffer from vestibular impairment, and should be diagnosed and rehabilitated accordingly.

## Data Availability Statement

The original contributions presented in the study are included in the article/supplementary materials, further inquiries can be directed to the corresponding author/s.

## Ethics Statement

The studies involving human participants were reviewed and approved by Regional Ethics Review Board (Study 1: Dnr LU178-07; Study 2: Dnr LU-2016/585), Lund University, Lund, Sweden. The patients/participants provided their written informed consent to participate in this study.

## Author Contributions

E-MM, EE, MM, and P-AF conceived the study design. E-MM, EE, and MM executed the data collection procedures. E-MM, EE, MM, AH, and P-AF analyzed and interpreted the results and contributed to the manuscript.

## Conflict of Interest

The authors declare that the research was conducted in the absence of any commercial or financial relationships that could be construed as a potential conflict of interest.
